# Acceptance and Commitment Therapy to Increase Resilience in Chronic Pain Patients: A Clinical Guideline

**DOI:** 10.3390/medicina58040499

**Published:** 2022-03-30

**Authors:** Maarten Moens, Julie Jansen, Ann De Smedt, Manuel Roulaud, Maxime Billot, Jorne Laton, Philippe Rigoard, Lisa Goudman

**Affiliations:** 1 Department of Neurosurgery, Universitair Ziekenhuis Brussel, Laarbeeklaan 101, 1090 Brussels, Belgium; maarten.moens@uzbrussel.be (M.M.); juliejansen@outlook.com (J.J.); 2 STIMULUS Research Consortium (Research and TeachIng Neuromodulation Uz Brussel), Vrije Universiteit Brussel, Laarbeeklaan 103, 1090 Brussels, Belgium; ann.desmedt@uzbrussel.be (A.D.S.); jorne.laton@uzbrussel.be (J.L.); 3 Center for Neurosciences (C4N), Vrije Universiteit Brussel, Laarbeeklaan 101, 1090 Brussels, Belgium; 4 Pain in Motion (PAIN) Research Group, Department of Physiotherapy, Human Physiology and Anatomy, Faculty of Physical Education and Physiotherapy, Vrije Universiteit Brussel, Laarbeeklaan 103, 1090 Brussels, Belgium; 5 Department of Radiology, Universitair Ziekenhuis Brussel, Laarbeeklaan 101, 1090 Brussels, Belgium; 6 Department of Physical Medicine and Rehabilitation, Universitair Ziekenhuis Brussel, Laarbeeklaan 101, 1090 Brussels, Belgium; 7 PRISMATICS Lab (Predictive Research in Spine/Neuromodulation Management and Thoracic Innovation/Cardiac Surgery), Poitiers University Hospital, 86021 Poitiers, France; manuel.roulaud@chu-poitiers.fr (M.R.); maxime.billot@chu-poitiers.fr (M.B.); philippe.rigoard@chu-poitiers.fr (P.R.); 8 AIMS Lab, Center for Neurosciences, UZ Brussel, Vrije Universiteit Brussels, 1090 Brussels, Belgium; 9 Nuffield Department of Clinical Neurosciences, University of Oxford, Oxford OX3 9DU, UK; 10 Department of Spine Surgery & Neuromodulation, Poitiers University Hospital, 86021 Poitiers, France; 11 Pprime Institute UPR 3346, CNRS, ISAE-ENSMA, University of Poitiers, 86360 Chasseneuil-du-Poitou, France; 12 Research Foundation—Flanders (FWO), 1090 Brussels, Belgium

**Keywords:** chronic pain, resilience, psychology, pain neuroscience education, cognitive-behavioural therapy

## Abstract

Chronic pain remains a very difficult condition to manage for healthcare workers and patients. Different options are being considered and a biopsychosocial approach seems to have the most benefit, since chronic pain influences biological, psychological and social factors. A conservative approach with medication is the most common type of treatment in chronic pain patients; however, a lot of side effects are often induced. Therefore, a premium is set on novel nonpharmacological therapy options for chronic pain, such as psychological interventions. Previous research has demonstrated that resilience is a very important aspect in coping with chronic pain. A more recent type of cognitive-behavioural therapy is Acceptance and Commitment Therapy, in which psychological flexibility is intended to be the end result. In this manuscript, current evidence is used to explain why and how a comprehensive and multimodal treatment for patients with chronic pain can be applied in clinical practice. This multimodal treatment consists of a combination of pain neuroscience education and cognitive-behavioural therapy, more specifically Acceptance and Commitment Therapy. The aim is to provide a clinical guideline on how to contribute to greater flexibility and resilience in patients with chronic pain.

## 1. Introduction

Pain management for chronic pain conditions that are incorporated within chronic primary pain often entail a multi- or interdisciplinary pain-management program, relying on a biopsychosocial approach [1], mostly with a focus on functional restoration [2]. For the management of long-term pain, the public opinion is currently in strong favour of self-management strategies as a first-line effective strategy, to engage patients to actively manage their own health status [3,4,5]. The safety and cost-effectiveness of self-management programs has been proven; nevertheless, effect sizes are small and not sustained in the long term [3,6]. Additionally, the limited efficacy in treating chronic pain with pharmacotherapy and the long-term side effects of these pharmacological treatment options [7,8] have put a premium on novel nonpharmacologic therapy options for chronic pain [9,10,11,12].

Since the 1960s, a number of psychological interventions for chronic pain have been developed based on psychosocial models [13]. First, the theoretical foundation for behavioural pain treatment [13] is provided by the operant-conditioning model [14,15]. Second, peripheral physiological models provide a theoretical foundation for relaxation training and biofeedback interventions [13]. Third, cognitive and coping models, first used to understand and develop treatments for chronic pain in the mid-1980s [16,17,18], provide the theoretical and empirical foundation for cognitive therapy and the group of cognitive-behavioural treatments that have emerged [13]. Finally, central-nervous-system neurophysiological models of pain, starting with the gate-control theory in the 1960s [19] and extending towards more complex models based on contemporary imaging studies [20,21], serve as neurophysiological explanations for the effects of many psychological interventions, as well as a rationale for psychological treatments that target brain processes and activity [13].

The intervention that will be proposed in this clinical guideline is Acceptance and Commitment Therapy (ACT) [22], which belongs to the cognitive-behavioural treatments and is an experiential therapy, based on clinical behaviour analysis [23]. ACT aims to decrease suffering and increase well-being through six core processes of change [24]. According to the contextual philosophy underlying ACT, the environment, behaviour, history and outcome of the behaviour are all part of the context and need to be considered while proceeding through the therapy [23]. In contrast to other models focused on reducing pain severity, ACT is based on the theory that attempts to modify certain aversive internal experiences, such as chronic pain, that may contribute to increased distress and interference [25,26]. ACT consists of awareness and nonjudgmental acceptance of all experiences, both negative and positive; identification of values; and appropriate action toward goals that support those values [27]. The main objective is to ameliorate functioning and decrease interference of pain with value-driven action whereby the mechanism is presumed to be acceptance [28]. Results in ACT with chronic pain have demonstrated that acceptance is associated with increased pain tolerance and better emotional, social and physical functioning [29]. ACT can increase psychological flexibility [30], which is defined as the capacity to persist or to change behaviour, including a conscious and open contact with discomfort and other discouraging experiences, guided by goals and values [22]. Psychological flexibility is thus regarded as a resilience factor among individuals with chronic pain [31]. The most straightforward definition of resilience is the ability to cope with shocks and to keep functioning (emotional and physical) in much the same kind of way [32]. It represents the ability to bounce back from adversity, whereby it is suggested that resilient individuals are more likely to engage in adaptive pain-coping strategies compared to nonresilient individuals [33].

More specifically in the context of pain, resilience is referred to as a set of adaptive responses to pain and pain-related life adversities [34]. When a person is exposed to an acute stress, she/he accesses physiological, affective, cognitive and social resources in response to the distress [34]. It is important to effectively and efficiently regain homeostasis upon the resolution of the challenge [34]. However, continued recurrent stress, such as chronic pain, makes it increasingly difficult to recover homeostasis [34]. A set of stable and modifiable factors exists in the intra- and interpersonal domains that may foster and/or hinder resilient functioning in chronic pain patients [34]. In a previous study [33], resilience was operationalised based on the results of the Profile of Chronic Pain: Screen questionnaire [35] whereby chronic pain patients were divided into the resilient sample if they scored ≥1 standard deviation above the average on pain severity and less than 1 standard deviation above the average on both the interference and emotional-burden subscales, and to the nonresilient sample otherwise. For patients with equal levels of pain severity (i.e., similar stressor), those belonging to the resilient sample presented with more positive self-talk, higher capacity for task persistence and higher levels of perceived control compared to those belonging to the nonresilient sample [33].

Results showed that ACT is efficacious for a number of conditions including anxiety, depression, substance use, pain and transdiagnostic groups and is generally superior to inactive controls (e.g., waitlist, placebo), treatment as usual and most active-intervention conditions (excluding cognitive-behavioural therapy) [36]. Specifically in the context of chronic pain, the use of ACT has drastically increased during the latest years, including in online format [37], with positive results on functioning [38,39] and improvements on health-related quality of life [40]. Despite the increasing number of studies that are exploring ACT, a clear perspective on how to provide ACT to chronic pain patients in clinical practice is still lacking. The aim of this clinical guideline is to provide a step-by-step guide on how to build resilience in a chronic pain population, through a multimodal treatment approach of eight sessions spread over a period of 8 weeks.

## 2. Acceptance and Commitment Therapy to Increase Resilience in Chronic Pain Patients

**Figure 1:**
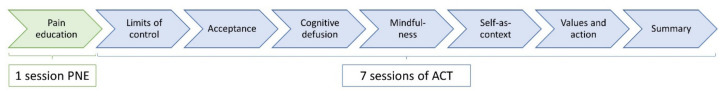
Overview of the Acceptance and Commitment Therapy Program, consisting of 1 session of Pain Neuroscience Education and 7 sessions of Acceptance and Commitment Therapy. Abbreviations. ACT: Acceptance and Commitment Therapy, PNE: Pain Neuroscience Education.

Figure 1 presents the full program, incorporating one session of Pain Neuroscience Education (PNE) and 7 sessions of ACT, each lasting one hour at a frequency of 1 h/week.

At the start of the educational program, all patients receive one session of Pain Neuroscience Education (PNE) [41,42], which is a biopsychosocial cognitive-based intervention. During PNE, the patient gains insights in the neurophysiology of pain, learns to reconceptualise pain, receives techniques to alter the beliefs regarding (chronic) pain, and gains insight in pain-related cognitions and coping strategies [43,44]. The education is scheduled at the start of the ACT educational program to avoid maladaptive attitudes, cognitions and behaviour in relation to pain, cognition and movement due to poor understanding of the principles underlying pain [44].

During the PNE session, all principles of an individual’s pain experience (i.e., biological, physiological and psychosocial processes) are explained in layman’s terms in combination with photos, metaphors and understandable sketches [43,44], with beneficial results on altering maladaptive cognitions, healthcare utilisation, pain and disability [44]. This first session lasts for approximately one hour, whereafter all patients receive an informational brochure with the same information [45] to maximise information retention [46].

ACT essentially consists of two core components, namely Acceptance and Commitment, distributed in six themes: acceptance, cognitive defusion, self-as-context, the here and now, values and committed action. Acceptance includes acceptance, cognitive defusion and self-as-context. The learning goal of this component is to deal with problems in a different way than usual. Instead of trying to have a solution for everything, patients learn how to carry unpleasant thoughts and feelings in a healthy way [47]. Commitment includes contact with the present moment, values and committed action. The learning goal of this component is to make an investment in yourself. This component handles about reflecting and exploring the topics that really matter in a person’s life [48].

We propose an organisational format of seven sessions (disregarding the first session with a focus on PNE) of 1 h, once per week. The main goal of ACT is to deal with problems in a different, more flexible way [48]. During the first ACT session, a brief introduction on resilience and the intervention is given. During this introduction, the therapist explains how resilience is the process of adapting well in the face of adversity, trauma, tragedy, threats or significant sources of stress, among which include serious health problems such as chronic pain [49]. Resilience involves “bouncing back” from these difficult experiences, but can also involve a profound personal growth [49].

The intervention involves Acceptance and Commitment therapy [22] and is explained as follows: ACT is a form of behavioural therapy with the goal of increasing psychological flexibility [48]. During the life course, people encounter all kinds of obstacles such as unpleasant thoughts, difficult emotions and unpleasant body sensations, which can be a prevention from realising dreams [48]. ACT provides different tools and techniques to deal with these unpleasant occurrences [48]. The aim is to stop being absorbed by negativity, such that more energy is left for valuable areas in life [48]. Thus, in the case of chronic pain, the goal is to reduce dominance of pain in person’s life through making patients’ responses toward symptoms more successful in relation to their own goals instead of focusing on symptom reduction [30]. This success is achieved by increasing psychological flexibility [30]. Psychological flexibility is defined as the capacity to persist or to change behaviour, including conscious and open contact with discomfort and other discouraging experiences, guided by goals and values [22] and is regarded as a resilience factor among individuals with chronic pain [31].

A detailed explanation about the content of the seven ACT sessions is provided below, with corresponding homework for each session (Supplementary Material SI). The homework assignments are important to continue working with the learned techniques at home, in order to reach the full potential of the intervention [48].

### 2.1. Session ACT 1

During the first session, the limits of control are examined. Using exercises and metaphors, the therapists explains that trying to control negative thoughts, feelings and circumstances is counterproductive [48]. The current strategies that the patient uses to cope with difficulties and unrealistic demands are explored [48]. Open dialogues between the patient and therapist demonstrate the futility of control-oriented strategies such as the suppression of thought and attempts to eliminate pain and/or distress [29]. When it is clear that control is not part of the solution but rather part of the problem, space can be created for more flexibility [48]. Avoidance is one of the strategies for dealing with difficulties in the context of chronic pain, meaning that the patient avoids unpleasant situations, difficult events, or certain activities. A short-term effect of avoidance is that the patient is not confronted with the unpleasant feelings, since the activity, event, etc., does not occur. This strategy of avoiding unpleasant feelings can be compared with throwing away a boomerang [48]. We know that a boomerang always returns to the person who threw it and can extend this principle to the avoidance strategy. If the patient keeps avoiding difficult situations, there is a chance that the problems will become even more difficult when the patient is confronted with them again at a later stage. The harder the patient tries to throw the boomerang away, the harder it will eventually return.

The theory behind this session is that cognitive rules make patients less sensitive to environmental contingencies [50]. Patients need to understand that cognitive rules may be either useful or problematic depending on the context, and they possess the flexibility to follow or abandon those rules depending on the situation [51]. Therefore, in ACT, small steps are taken to help patients to shape behaviour according to what the environmental contingencies suggest is most effective, taking into account that all rules can change depending on the situation [51].

### 2.2. Session ACT 2

The aim of this second session is to offer acceptance as an alternative to resistance [48]. Within acceptance, inner experiences are embraced, while they are occurring as ongoing inner experiences [51]. This is an active action, and is not the same as tolerance since acceptance involves a free choice [51]. The difference between pain and suffering is explained, whereby pain is caused by immediate circumstances and is unavoidable [48]. If there is no direct cause, we speak of suffering [48]. Pain is often turned into suffering because we want to control and avoid pain [48]. During this session, several examples can explain the difference between pain and suffering, among which includes the example of playing tug-of-war on the edge of a ravine with a monster [48]. The patient imagines that he/she will fall off the edge into the ravine if he/she is not pulling hard enough. Nevertheless, the harder the patient pulls, the harder the monster pulls. Thus, the question is, how does the patient play tug-of-war in his/her daily life? Does this strategy solve the reported problems? What would happen if the patient stopped fighting and released the rope? The idea here is that the monster keeps screaming, but the patient does not need to fight it anymore. If the patient stops the struggle, then all that is left is the real pain and not the suffering. This is the point where willingness comes in, indicating that it is not about feeling better, but learning to feel better.

### 2.3. Session ACT 3

This session is dedicated to cognitive defusion. Cognitive defusion can be thought of as reducing the literal meaning of inner experiences so that thoughts are purely experienced as thoughts, feelings are just feelings, and bodily sensations are just bodily sensations [48,51]. Cognitive defusion is the contrary of cognitive fusion, where inner experiences have a lot of power on the actions of a patient and are taken literally [51]. Cognitive defusion and cognitive fusion are both useful when they can be flexibly applied to different situations [51]. This skill can help patients to distance themselves from the contents of their thoughts [48]. It can give more space to decide for themselves whether they do something with the thought, or let it pass [48]. Distancing from thoughts can create space for mindfulness, another look at the self, values and committed action [48]. The example of a waterfall of thoughts is one of the techniques that the therapist can use in this session [48]: *“The mind produces a waterfall of thoughts. You can do two things. Either you can stand under the waterfall and let yourself be swept away by the thoughts, or you can try to stand behind the waterfall to observe your thoughts. From behind the waterfall, you are watching your thoughts, but you are no longer being pulled along by them.”*

Previous research demonstrated that cognitive defusion reduces discomfort and believability more than comparison approaches [52].

### 2.4. Session ACT 4

Mindfulness is explained in the fourth session as to be consciously open to everything that goes through your body and to everything that happens around you [48]. Being present is when we experience our inner experiences and actions in the environment at this time, namely as occurring now, in contrast to actions from the past or future [51]. Being present is considered as a flexible and voluntary attention to internal and external events, without attachment to evaluation or judgment [51].

The strategy underlying mindfulness-based interventions is to emphasise nonjudgment, meaning that physical pain and emotional suffering are detached from each other [53]. During this session, a number of exercises are performed together with the patient, among which includes a body scan [48]. Body scans are a core component of mindfulness meditation. They involve attention on the present moment through observing the breath and bodily sensations, while becoming aware of, and accepting without judgement, any thoughts and feelings that may arise [54]. A 10 min body scan is already able to reduce pain-related distress ratings and perceived interference of pain in social relations [54]. Another exercise is a 3 min mindfulness breathing intervention (awareness of breath), resulting in lower levels of stress, lower pain intensity and pain unpleasantness [55]. 

### 2.5. Session ACT 5

During the fifth session, the thoughts patients have about themselves are explored [48]. The conceptualised self is the “you” that is created, based upon self-evaluations [51]. In other words, this is a representation of what we believe ourselves to be [51]. Patients attempt to protect and retain that conceptualisation, even in situations where it results in ineffective action [51]. In ACT, we develop a sense of self as context, where the self is the place of awareness or perspective taking that allows internal and external events to be experienced from “I/here/now”, without being defined by those events [51]. From this sense of self as the context where inner experiences occur, we can voluntarily decide to adhere to our senses of who we are, or not [51]. This context is developed by providing mindfulness exercises, metaphors and experiential processes [52]. Patients are challenged to see their thoughts and feeling as pieces on a chess board [48]. Positive thoughts and feelings are represented as white chess pieces, while negative thoughts and feelings are denoted by black pieces. The patient is encouraged to see this battle that is taking place in his/her head. What is the position of the patient in this battle (i.e., the white or the black pieces)? We usually want to represent the white pieces because we want white (the positive) to win. Unfortunately, this is not how it works in practice. Black is a formidable opponent and will sooner or later lay a trick on you again. As such, our head is full of both positive and negative thoughts and feelings. So as a person, we obviously represent both the white and the black pieces, since we are both of them. As such, we are fighting with white against ourselves. The patient is encouraged to reflect about this situation. Moreover, the helicopter view could be used to let the patient think about himself/herself even more as follows: *“Everyone looks at the world from their own point of view. You could compare yourself to a video camera that films your inner world (thoughts, feelings and physical experiences) as well as the environment. The disadvantage of this particular point of view is that we cannot see ourselves. When you are the camera, you cannot film yourself. Nevertheless, this would be an appealing thing to do, resulting in a lot of self-knowledge. Try to imagine that you are looking at yourself from a helicopter. From this position you could see, for example, that your head is full with thoughts, or that you are easily irritated. Because your own camera is focused on the environment, you cannot see what you are doing yourself. Shall we try sitting in the helicopter? What do you see when you look at yourself?”*

### 2.6. Session ACT 6

This session mainly consists of exploring the values in patients’ lives [48]. Values are chosen qualities of purposive action that can be instantiated moment by moment [52]. ACT uses a variety of exercises to help a patient choose life directions in various domains [52]. Assistance in clarifying patients’ values should help make it more likely that they will approach stimuli that originally fostered avoidance [51]. If patients know what they really find important, they know in which areas of their life they want to invest [48]. In that way, they can make choices based on desires that are truly of them instead of those imposed by their intellect and/or environment [48]. The therapist can ask the patient about dreams. Afterwards, explore what is appealing in each dream and what is so beautiful about it, to gain insight in underlying values.

**Figure 2:**
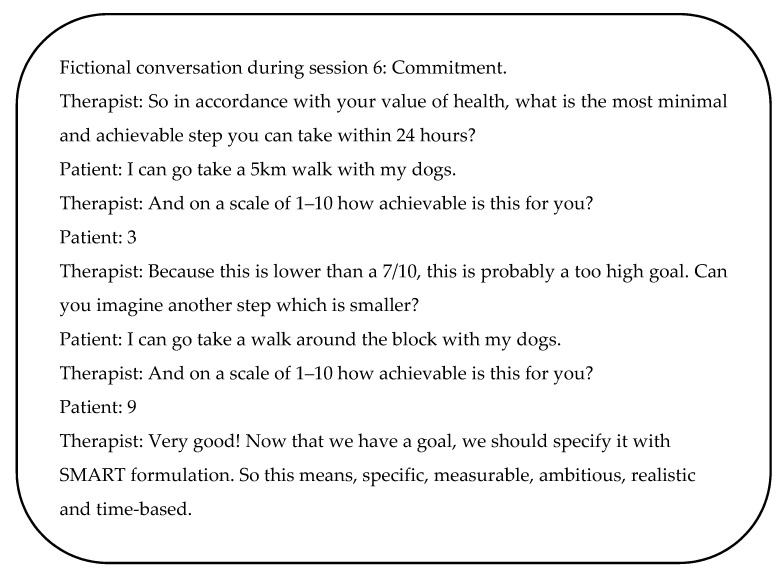
Example of a conversation between therapist and patient related to goal defining. Abbreviations. A: achievable, M: measurable, R: relevant, S: specific, T: time-bound.

ACT also promotes the development of larger and larger patterns of effective action linked to chosen values [52]. Committed action is the continuous redirection of behaviour with the aim of developing large patterns of flexible and effective behaviour linked to a specific value [51]. It involves defining personal goals and acting upon those goals, while practicing the other ACT strategies, with the ultimate goal of building larger patterns of values-oriented action [51] (Figure 2). To denote the difference between two behavioural styles, the metaphor of a football match can be used [48]. There are essentially two groups of people present at a football match: the spectators and the players on the football pitch. The people in the stands are busy watching the match: they analyse the game and try to find out what is happening, and shout instructions and comments to the players and referee. Despite the efforts that the visitors put into the match, we know that this probably has very little influence on the course of the match. We can compare these people to the people on the football pitch: the players. The communication of the players on the pitch is all about improving the match. This must then be translated directly into action. In order to play well, they do their best, with full commitment, to advance the game. Their conversations and the resulting actions have a great influence on the game and are very important. How does the patient stand in his/her life: sitting on the stands watching and judging or on the field working for a good outcome?

Patients are encouraged to set short- and long-term goals that are consistent with their values [29]. Unlike values, which are constantly instantiated but never achieved as an object, concrete goals can be achieved. Therefore, ACT protocols most likely incorporate therapy sessions related to short, medium and long-term goals [52]. 

### 2.7. Session ACT 7

In the seventh and last session, all six core components of ACT are recapitulated (Table 1). The core ACT processes are both overlapping and interrelated [52]. The processes support each other and all aspects target psychological flexibility: the process of contacting the present moment fully as a conscious human being and persisting or changing behaviour in the service of chosen values [52]. The therapist explains the relation between the core ACT processes as learning to dance [48]. Learning to dance starts with repeated practice of the basic elements of the dance: lightly leaning on the ball of your foot, the basic steps, keeping your balance and keeping time. Once you have gained some skill in each of those aspects, you will learn how to put them together into a coherent pattern. As you practice, you will find that you are increasingly able to dance smoothly and quickly and to switch effortlessly between the different skills. You learned these skills because you wanted to learn to dance, not because you wanted to be able to do each skill separately. It is only when you play together that the full value of all these skills is revealed. Thus, we define ACT as a psychological intervention based on behavioural psychology that applies mindfulness and acceptance processes, and commitment and behaviour-change processes, to enhance psychological flexibility (considered as a resilience factor) [52]. This resilience enables patients to deal flexibly with the problems that come in their way, so that they do not allow themselves to be led by them, but continue to fill in their life based on what is really important to them [48]. During the session we reflect on the program and focus on how ACT can be integrated in the daily life of the patients [48].

## 3. Monitor Resilience

During the ACT sessions, the therapist is helping the patient to accept the presence of chronic pain and building on strategies (as presented in each of the sessions) to ultimately develop greater psychological flexibility [53], considered as a resilience factor in this population [31]. Resilience should be considered as a continuum ranging from low resilience to high resilience (i.e., strong capacity to recover) and extremely high resilience, reflecting the ability of a person to reach a superior level of functioning after an adverse of stressful event [56], rather than as a binary variable of possessing this capacity or not. Several interventions are suggested to be effective as resilience training among which cognitive behavioural therapy and mindfulness-based interventions [56], pointing towards the value of self-report resilience scales. Nowadays, several self-reporting measures of resilience are available among which include the Brief Resilience Scale [57], the Connor-Davidson Resilience scale [58] or the Scale of Protective Factors [59].

## 4. Practical Considerations

ACT is a transdiagnostic form of therapy, which means it is not developed specifically for a particular diagnosis or disorder [48]. ACT focuses on learning to deal with complaints functionally, so that difficult but valuable experiences are no longer avoided [48]. The processes of ACT are widely applicable, with different psychological complaints and problems and therefore applicable in a very broad range of settings [48]. The currently proposed intervention was not offered as part of an interdisciplinary integrated pain-rehabilitation program; rather, it was designed to be appropriate for use in primary care. Qualified programs of ACT should consist of six core operational processes: acceptance, cognitive defusion, being present, self-as-context, values and committed action [52]. Both of the two groupings—mindfulness and acceptance processes, and commitment and behaviour change processes—are required to be included in the intervention [60]. Six sessions are recommended as a lower limit, to ensure that all ACT components are properly addressed [48]. It is advisable to leave a minimum of one week between sessions so that patients can practice sufficiently with the material [48]. In this program, one session of PNE and seven sessions of ACT are outlined. As such, it was much less intensive than the intervention used in some investigations (e.g., 12 h total over 8 weeks vs 7.5 h per day over a 3- to 4-week period [61]). In this program, an introduction of limits of control is added, as well as a summary of all components [48]. Moreover, values and commitment are combined into one session [52].

An important characteristic of the intervention are the home assignments. In this intervention, techniques are learned during the sessions whereby patients are expected and motivated to continue working with these concepts at home [48]. If the translation towards implementation in daily practice does not happen, the intervention may not be fully effective [48]. Handouts can be a valuable addition to ACT, so that patients can reread the exercises later and try them out at home [48]. Another way of helping patients to think about the concepts they have learned during the ACT sessions is to provide them short summary videos in the time period between sessions [61]. As the six core components become powerful enough that they are continued outside of session, it is hoped that the patient can contact actual contingencies in the world and learns how to function better within them [51].

Presumably, the ACT sessions are best provided by a therapist who is specialised in ACT. It is important that the practitioner has a good understanding of ACT and its core processes. Learning how to become and be an ACT therapist can be achieved by following training or reading manuals [62]. ACT has developed quite an extensive list of core therapist competencies that therapist should strive to acquire [62]. The therapeutic relationship is very important in ACT and helpful suggestions may be necessary on how to approach difficult situations that can be experienced with clients when applying ACT [62]. ACT training courses are offered by various institutions and usually consist of several days (3–8 days), which includes sessions of supervision and intervision. For the PNE sessions, a transdisciplinary manner of providing the session with shared input from a psychologist and physiotherapist would be highly beneficial. Physical therapists have extensive knowledge concerning the anatomy of the body and the influence of cognitions on biomedical factors, while psychologists are more familiar with handling persistent maladaptive cognitive factors and encouraging a behavioural change in complicated situations such as chronic pain [43].

ACT is particularly suitable for use in a group format [48]. The program is outstanding for interactive use in a group and group members can help each other with the various themes by telling each other about how they have approached them [48]. If, as a therapist, you want to work with ACT in groups, it is advisable to have experience with group therapy [48]. The recommended group size is 8–12 participants for optimal group dynamics [48].

## 5. Conclusions

The present perspective article explained why and how a comprehensive treatment, consisting of PNE and ACT, for individuals with chronic pain can be applied in clinical practice. In future studies, it might be interesting to further explore the effectiveness of existing psychological-treatment interventions in the chronic pain population, and more specifically whether this treatment proposal, consisting of PNE and ACT, can contribute to greater flexibility and resilience in chronic pain patients.

## Figures and Tables

**Table 1:** Content of Acceptance and Commitment sessions, with corresponding home assignments.

ACT Sessions	Content of Session	Home Assignment
Session 1	Limits of control	Taking stock of strategies for dealing with difficulties. Identify the demands that are imposed.
Session 2	Acceptance	Insight in pain and suffering. Practising in the readiness to have negative emotions and thoughts. Break through the demands that are imposed.
Session 3	Cognitive defusion	Exercises to create distancing from thoughts.
Session 4	Mindfulness	Mindfulness exercises.
Session 5	Self-as-context	Taking stock of the role of our self in different contexts.
Session 6	Values and committed action	Create an action plan on how to put values into practice.
Session 7	Summarisation	Create an action plan on how to integrate ACT in the daily life.

## Data Availability

Not applicable.

## Supplementary Materials

The following supporting information can be downloaded at: https://www.mdpi.com/article/10.3390/medicina58040499/s1, Supplementary Material SI: Homework assignments for Acceptance and Commitment Therapy.
