# Supplementary material for: Acceptance and Commitment Therapy to Increase Resilience in Chronic Pain Patients: A Clinical Guideline
Source: Medicina (Kaunas). 2022 Mar 30;58(4):499. doi: 10.3390/medicina58040499 (PMC9025890; doi:10.3390/medicina58040499)
Supplement: Supplementary file 1 [file medicina-58-00499-s001.zip › medicina-1573265-supplementary.pdf]

Supplementary material SI: Homework assignments for Acceptance and Commitment Therapy

Session ACT 1

At the end of the first ACT session, two homework assignments are given. The first involves taking stock of strategies for dealing with difficulties. Patients write down an unpleasant experience, combined with the strategy to cope with this unpleasant experience, the short-term results, long-term results and the price they paid for this strategy. The second task is to identify the demands that are imposed.

Session ACT 2

The homework assignments focus on insights in pain and suffering, and practicing in the readiness to have negative emotions and thoughts. Another exercise is to break through the demands that are imposed. As such, the patient is asked to reflect on an unpleasant thought, feeling or experience and tries to accept that it is there. The patient explicitly thinks about this experience and does not try to get rid of this feeling. Over time, the pain will become easier to handle, which reduces its impact on the patient's life.

Session ACT 3

The homework assignments include exercises to create distance from thoughts. Patients try, for example, to see their thoughts as subtitles in a movie. A subtitle is at the very bottom of the film, so it does not take up all the space. You can choose to give it attention, but most of all you want to watch the film. Additionally, patients also practice in doing the opposite of what their mind says. Patients perform activities while saying out loud that they cannot do them (e.g., to walk while saying that you are paralysed, ride a bicycle while saying that you cannot, and breathe when you say you cannot breathe). Negative thoughts can only affect us if we believe them. This week, patients experience that with the craziest thoughts, they can still do what they really want to do.

Session ACT 4

Mindfulness is a skill that everyone can acquire, so it is important that time is set aside for practicing. For that reason, the homework is that the patients have to practice a lot to learn mindfulness.

Session ACT 5

This week, patients are instructed to regularly pause to consider the following question: "Am I now acting on the basis of a certain image that I have of myself and is this the way I want to deal with the situation?" If the answer to the last question is no, then consider how you would like to deal with this situation and then do so. In this way the patient is working on recognizing, as well as breaking through the automatic patterns of life.

Session ACT 6

ACT protocols most likely incorporate therapy sessions and homework assignments related to short, medium and long-term goals. This is also the home assignment of this session, namely to create an action plan on how to put values into practice. Additionally, patients are instructed to give attention to their values. The following instructions can be provided to the patient: "Take 15 min every day to dream freely about your life. For example, what would you like in your relationship? Where would you like to live? What is your dream job? What would you still like to do or undertake? Additionally, take a daily picture of a moment that is meaningful to you. In this way you gain insight into the values that are present in your life at this moment."
